# One of the First Cases with PIK3CA-related Overgrowth Spectrum (PROS) in Saudi Arabia: A Case Report and Literature Review

**DOI:** 10.7759/cureus.6586

**Published:** 2020-01-07

**Authors:** Saleem A Alsaedi, Oday Qurashi, Mohammed Bajunaid, Abdullah A Altalhi, Aiman M Shawli

**Affiliations:** 1 Medicine, College of Medicine, King Saud Bin Abdulaziz University for Health Sciences, Jeddah, SAU; 2 General Pediatrics and Pediatric Genetics, King Abdulaziz Medical City, Jeddah, SAU

**Keywords:** pik3ca, mcap, pros, overgrowth syndrome

## Abstract

PIK3CA-related overgrowth spectrum (PROS) is an umbrella that includes a broad range of rare disorders, ranging from isolated digit enlargement to extensive overgrowth of the limbs, abdomen, or brain. One of these disorders is megalencephaly capillary malformation polymicrogyria syndrome (MCAP), which is characterized by cutaneous capillary malformations, megalencephaly, cortical brain malformations, abnormalities of somatic growth with body and brain asymmetry, developmental delay, and characteristic facial dysmorphism. The diagnosis of PROS syndrome is based on the clinical features of a patient and confirmed by a pathogenic variant in one PIK3CA allele in a biopsy of the affected tissue. However, MCAP may be diagnosed by testing a blood or saliva sample. The management of patients with MCAP syndrome includes evaluation after the initial diagnosis, treatment of manifestations, and surveillance for potential complications. To date, there is no curative treatment for patients with MCAP syndrome. Therefore, reporting such cases will help us understand them and thus develop an appropriate treatment for them.

Our patient was a 46-month-old boy, who is diagnosed with MCAP syndrome. The diagnosis was based on clinical presentation, imaging studies, and whole-exome sequencing (WES). Clinically, the patient had speech and developmental delay, macrocephaly, joint hyperlaxity, unsteady gait, and subtle dysmorphic facial features. The facial features include low-set ears, frontal bossing, depressed nasal bridge, and bilateral esotropia. MRI studies showed megalocephaly, bilateral perisylvian polymicrogyria, bilateral peri-regional, high T2 signal intensities, and cerebellar tonsil ectopia with crowding of the posterior fossa. Finally, the diagnosis was confirmed by WES, which detected changes in the PIK3CA gene. The patient is on overgrowth protocol for PIK3CA, which includes alpha-fetoprotein and abdominal ultrasound every three months until the age of eight years.

To the best of our knowledge, this is one of the first cases of PROS in Saudi Arabia, which illustrates the classical findings of MCAP syndrome. Further studies and investigations on PROS syndrome are needed to aid in making a definitive classification and treatment of such complex and rare diseases.

## Introduction

Segmental overgrowth is excessive growth affecting only some parts of the body. It is almost invariably asymmetrical and a feature of a heterogeneous group of rare disorders, which often carry a significant burden of morbidity and mortality. Affected patients commonly develop distorting or restricting overgrowth, leading to marked functional impairments.

Researchers have identified somatic activating mutations in components of the phosphatidylinositol-3-kinase (PI3K)-PTEN-AKT-mTOR signaling pathway in many patients with different forms of segmental overgrowth. PIK3CA encodes p110α, the catalytic subunit of phosphatidylinositide-3-kinase (PI3K). This signaling enzyme is critical for the growth, survival, and metabolism of most cells and is one of the most commonly mutated genes found in cancers. PIK3CA-related overgrowth spectrum (PROS) is an umbrella that covers a wide spectrum of clinical phenotypes, ranging from isolated digit enlargement to extensive overgrowth of the limbs, abdomen, or brain, often in association with vascular malformations [1].

To date, the deformity of overgrowth syndromes caused by somatic PIK3CA mutation includes fibroadipose hyperplasia or overgrowth (FAO), hemihyperplasia multiple lipomatosis (HHML), congenital lipomatous overgrowth, vascular malformations, epidermal nevi, scoliosis/skeletal and spinal (CLOVES) syndrome, fibroadipose infiltrating lipomatosis, megalencephaly-capillary malformation (MCAP or M-CM), dysplastic megalencephaly (DMEG), and isolated macrodactyly [2].

The diagnosis of PROS syndrome is based on the clinical features of a patient and is confirmed by a pathogenic variant in one PIK3CA allele in a biopsy sample of the affected tissue. However, MCAP may be diagnosed by testing a blood or saliva sample. Nevertheless, failure to detect a PIK3CA mutation does not rule out a clinical diagnosis of PROS in individuals with suggestive features, as this may be due to sub-optimal tissue biopsies or low-level mosaicism. The management of patients with PROS syndrome includes evaluation after the initial diagnosis, treatment of manifestations, and survivance for potential complications [2-3].

The current rapid identification and study of new patients with mutations in the PI3K/AKT pathway mean that a definitive reclassification is likely to occur when the full disease spectrum and any genotype-phenotype correlations have been discovered.

In this case report, we discuss the case of a 46-month-old boy with macrocephaly, developmental delay, and idiopathic hemihypertrophy. After further workup, the patient was diagnosed with PROS, specifically MCAP.

## Case presentation

The patient was a 46-month-old Saudi boy. He was delivered via cesarean section in view of his large head detected through prenatal ultrasound at term. He weighed 4.63 kg, measured 55 cm in length, and had a head circumference of 41 cm. He was discharged with his mother without admission to the neonatal intensive care unit (NICU). The mother had no antenatal or postnatal problems, and she had no prior medical illness. The head circumference was very large at birth, above the 98th centile. The patient was being reviewed regularly by ultrasound due to the large head size, which was normal initially in the neonatal period. There was no consanguinity and no family history of a similar condition.

**Figure 1:**
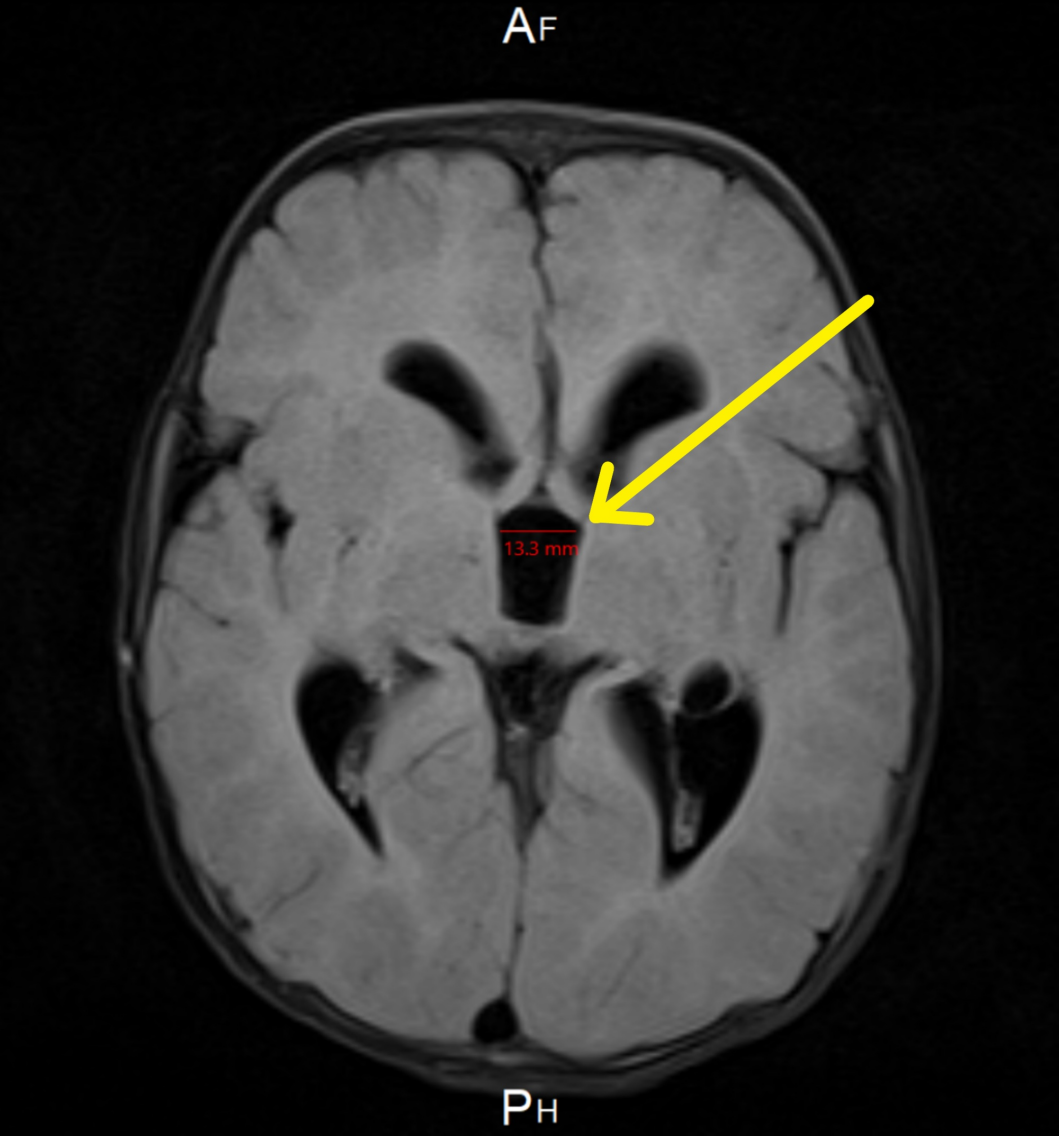
Brain MRI showing dilation of the third ventricle

**Figure 2:**
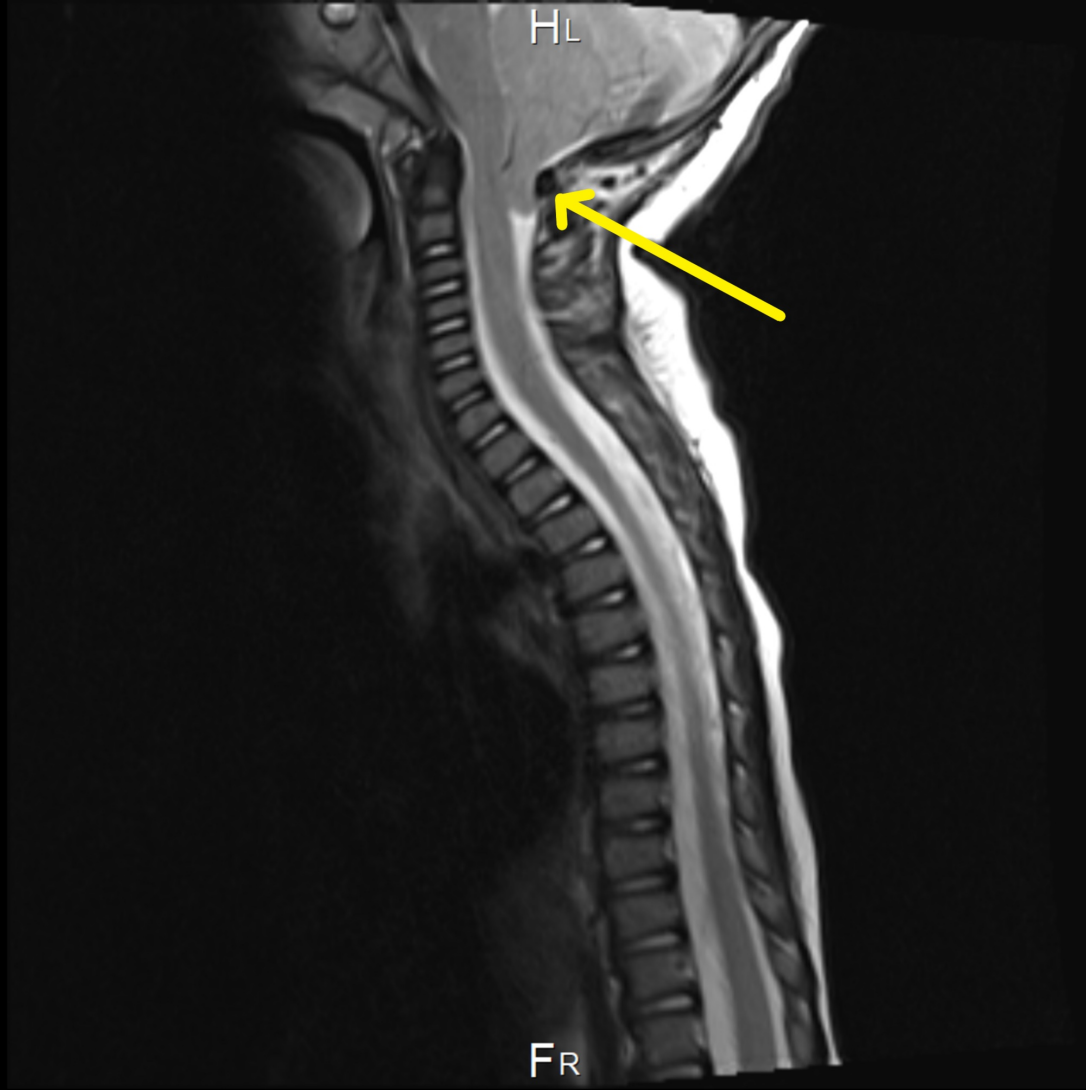
Whole spine MRI showing a significant descent of the cerebellar tonsils through the foreman magnum to the left of C2

At the age of six months, magnetic resonance imaging (MRI) showed intracranial hypotension with engorgement of the venous sinus and mild flattening of the pons. Tonsillar descent, third ventricle dilation, and white matter changes were also noted (Figure 1). Two months later, the patient underwent a whole spine MRI, which was incomplete, as the patient was fully awake during the study. The study redemonstrates the significant descent of the cerebellar tonsils through the foreman magnum to the left of C2. Also, the visualized part of the dural venous sinus at the posterior fossa was significantly distended (Figure 2). The abdominal ultrasound (US), which was done at the age of eight months to rule out organomegaly, was normal (Table 1). Parents of the patient kept following up with the neurology, orthopedic, metabolic, and genetics teams in King Abdulaziz Medical City in Jeddah.

**Table 1:** Imaging studies CBD: common bile duct

Computed Tomography of the Brain	
Report	Date
- Ventricular dilation - No intracranial hemorrhage, infarct, or midline shift. - Tonsillar herniation in the posterior fossa and sagging of the brainstem is noted. - Posterior periventricular hypodense white matter is identified. - The orbital images were unremarkable - Minimal opacification in the paranasal sinuses with mastoid air in the right middle ear.	09/02/2015
Abdominal Ultrasound	
Report	Date
- The liver size is 8.9 cm with homogenous echogenicity and no focal lesion. - No intrahepatic or extrahepatic bile duct dilation - The gallbladder is partially distended with normal wall thickness - CBD within the normal range, measuring about 0.17 cm. - Portal vein is patent and measuring about 0.7 cm - The spleen is of normal size, measuring about 5.8 cm with no focal lesion. - Both kidneys are normal in size and echogenicity without a focal lesion or hydronephrosis. - The visualized part of the pancreas was unremarkable.	9/11/2014
- The liver size is 11.5 cm with homogenous echogenicity and no focal lesion. - Unremarkable appearance of the gallbladder and CBD - CBD within the normal range, measuring about 1.7 cm. - Portal vein is normal and measuring about 0.7 cm - The spleen is of normal size, measuring about 7 cm with no focal lesion. - Both kidneys were of normal size and showed preserved corticomedullary differentiation without stones, cystic masses, solid masses, focal lesion or hydronephrosis. - The visualized part of the pancreas was unremarkable.	09/08/2016
- The liver size is 11.3 cm with homogenous echogenicity and no focal lesion. - No intrahepatic bile duct dilation - The gallbladder was collapsed - CBD within the normal range, measuring about 1.7 cm. - Portal vein is normal and measuring about 0.7 cm - The spleen is of normal size, measuring about 8.09 cm with no focal lesion. - The right kidney measures 7.9 x 2.8 cm with cortical thickness of 1.17 - The left kidney measures 7.50 x 4.23 cm with cortical thickness of 1.38 - Both kidneys showed preserved corticomedullary differentiation without stones, focal lesion or hydronephrosis. - The visualized part of the pancreas was unremarkable.	19/01/2017
- The liver size is 11.7 cm with preserved parenchymal echogenicity with no definite lesion. - No intrahepatic bile duct dilation - The gallbladder was normal - CBD within the normal range, measuring about 0.1 cm. - Portal vein is normal and measuring about 0.8 cm - The spleen is of normal size, measuring about 9 cm - Both kidneys and the visualized part of the pancreas were unremarkable - The urinary bladder was underfilled.	19/09/2017
Brain MRI	
Report	Date
- Intracranial hypotension with engorgement of the venous sinus and mild flattening of the pons. - Crowding of the posterior fossa and descent of the cerebellum inferiorly with mild herniation of the cerebellum. - Mild hydrocephalus is noted - Visualized parts from the orbits are unremarkable - The third ventricle is mildly dilated and is measuring around 1.3 cm in greatest diameter.	29/09/2014
- Evidence of macrocephaly of the brain - Bifrontal and perisylvian polymicrogyria with areas of prominent Virchow Robin spaces - Unchanged cerebellar tonsil ectopia with crowding of the posterior fossa. - Bilateral peri-regional high T2 signal intensities were noted. - No acute hemorrhage, abnormal enhancing region or meningeal enhancement.	22/8/2017
Whole Spine MRI	
Report (The examination is incomplete, as the patient is fully awake during the study. Few sagittal acquired images are obtained on the T1 and T2 weighted images).	Date
- Significant descendant of the cerebellar tonsils through the foreman magnum to the left of C2 - The partially visualized spinal cord demonstrates normal caliber with no evidence of syrinx - No significant intramural lesions or intradural masses - The visualized vertebral bodies are unremarkable - The conus terminates at the level of lower L2 and no evidence of intraspinal fatty lesions - Note is made to the significantly distended visualized part of the dural venous sinus at the posterior fossa.	10/11/2014

**Figure 3:**
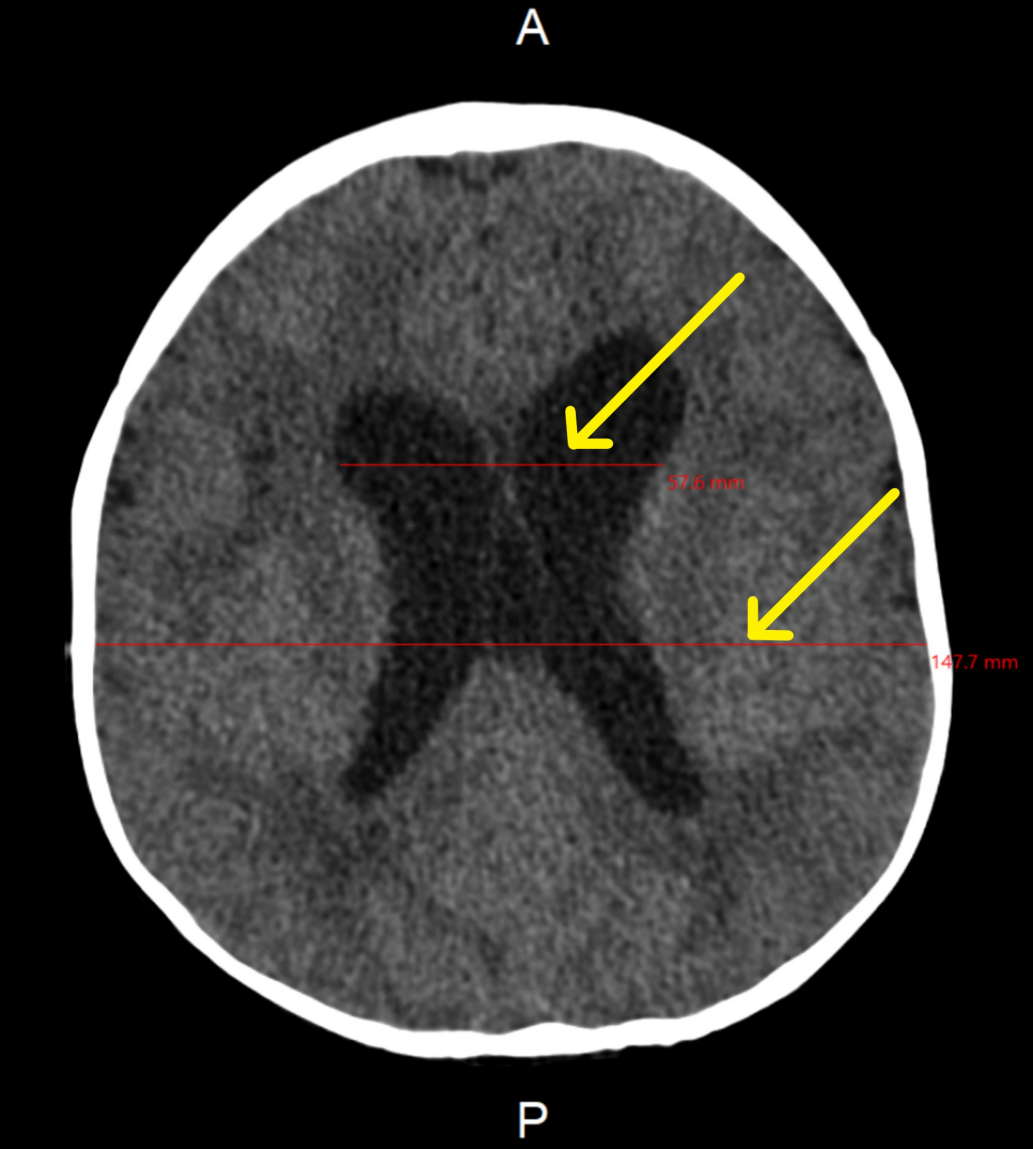
Brain CT showing ventricular dilation

At the age of 11 months, the patient was sent for computed tomography (CT) to assess the need for a ventriculoperitoneal (VP) shunt. However, the CT showed decreased hydrocephalus, so there was no need for a VP shunt (Figure 3). The patient was also seen by the ophthalmologist who confirmed that the optic disc was normal, with no pupillary edema.

At the age of 28 months, the patient underwent orchidopexy to bring down the left undescended testis from the intracanalicular level to the mid-scrotal level. He was discharged with no complications, and he was on a course of oral acetaminophen (24 mg/ml) (250 mg) (qid) for 10 days. A follow-up abdominal US was performed to reassess organomegaly, which revealed an increase in liver and spleen span with no evidence of a focal lesion (Table 1). Full metabolic workup and other laboratory tests were done and were normal (Table 2).

**Table 2:** Laboratory tests *Actual result was not found; it was taken from a medical report. †Actual result and date were not found; it was taken from a medical report. ‡Actual result was not found; it was taken from a medical report. The test was repeated in 2014, 2015, and 2016. TSH: thyroid-stimulating hormone; TRPG: tricuspid regurgitation peak gradient

Lab Results							
19/05/2016	09/08/2016	08/11/2016	02/03/2017	06/07/2017	25/07/2017	09/01/2018	Analytes
		10.6	10.1	10.3	10.1	10.9	Hemoglobin (gm/dL)
		4.4	3.6	2.6	3.6	3.6	White blood cells (×10^9^/L)
		290	228	153	195	248	Platelets count (×10^9^/L)
			30.5	39.5	29.5	33.5	Hematocrit (%)
				40		38	Partial thromboplastin time (seconds)
		0.9		1.0		1.1	international normalized ratio
				3.5			Urea nitrogen (mmol/L)
35							Albumin (g/L)
		42					Creatinine kinase (IU/L)
		2.27					TSH (mIU/L)
		10					Free T4 (pmol/L)
	1	Normal*					Alpha fetoprotein (ng/mL)
Normal†							N-aspartate (IU/L)
Normal†							TRPG (mm Hg)
Normal†							Lactic acid (mmol/L)
		Normal*					Bone profile
		Normal‡					Serum amino acid (mmol/L)

**Figure 4:**
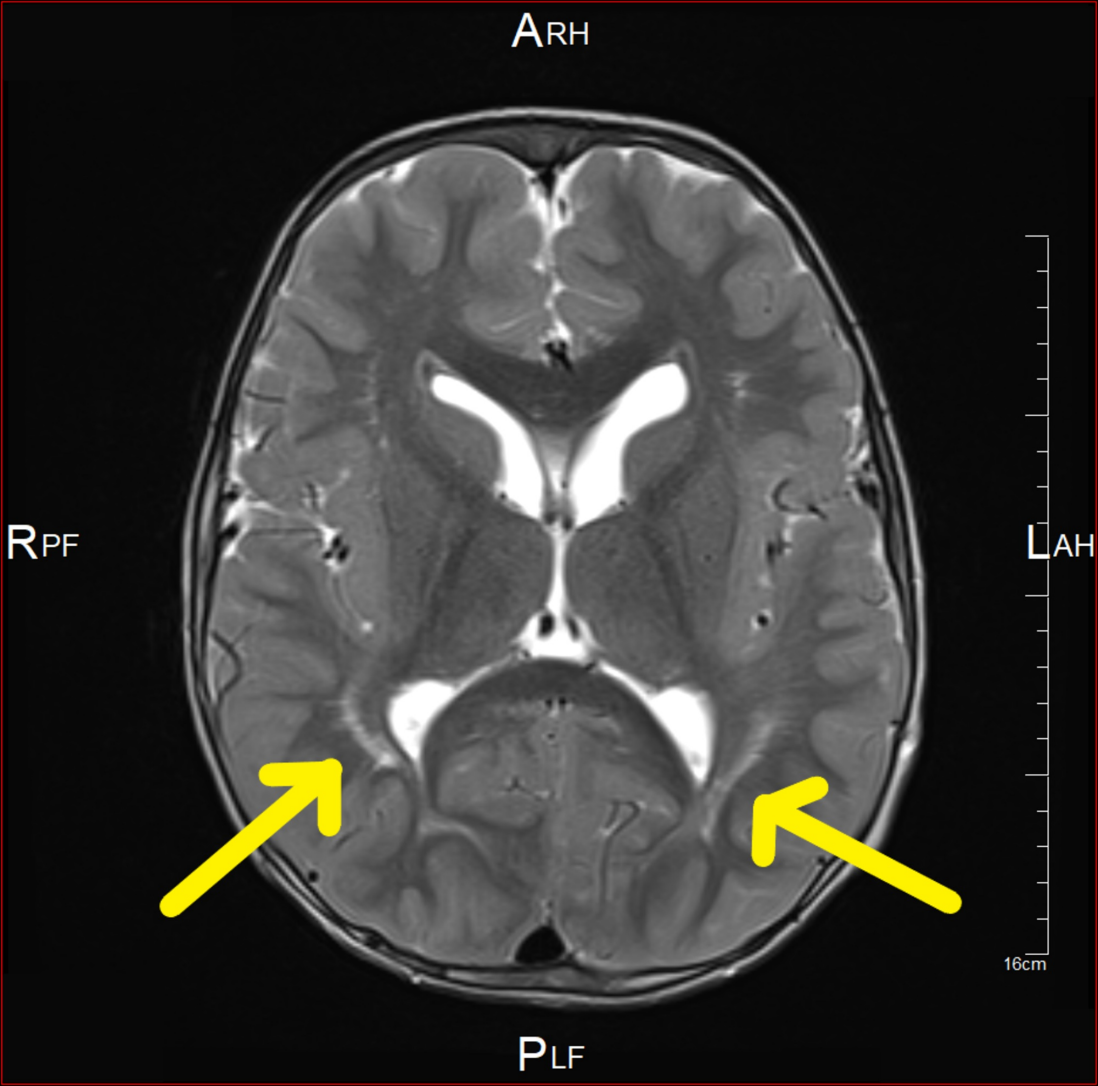
Brain MRI showing bilateral peri-regional high T2 signal intensity

**Figure 5:**
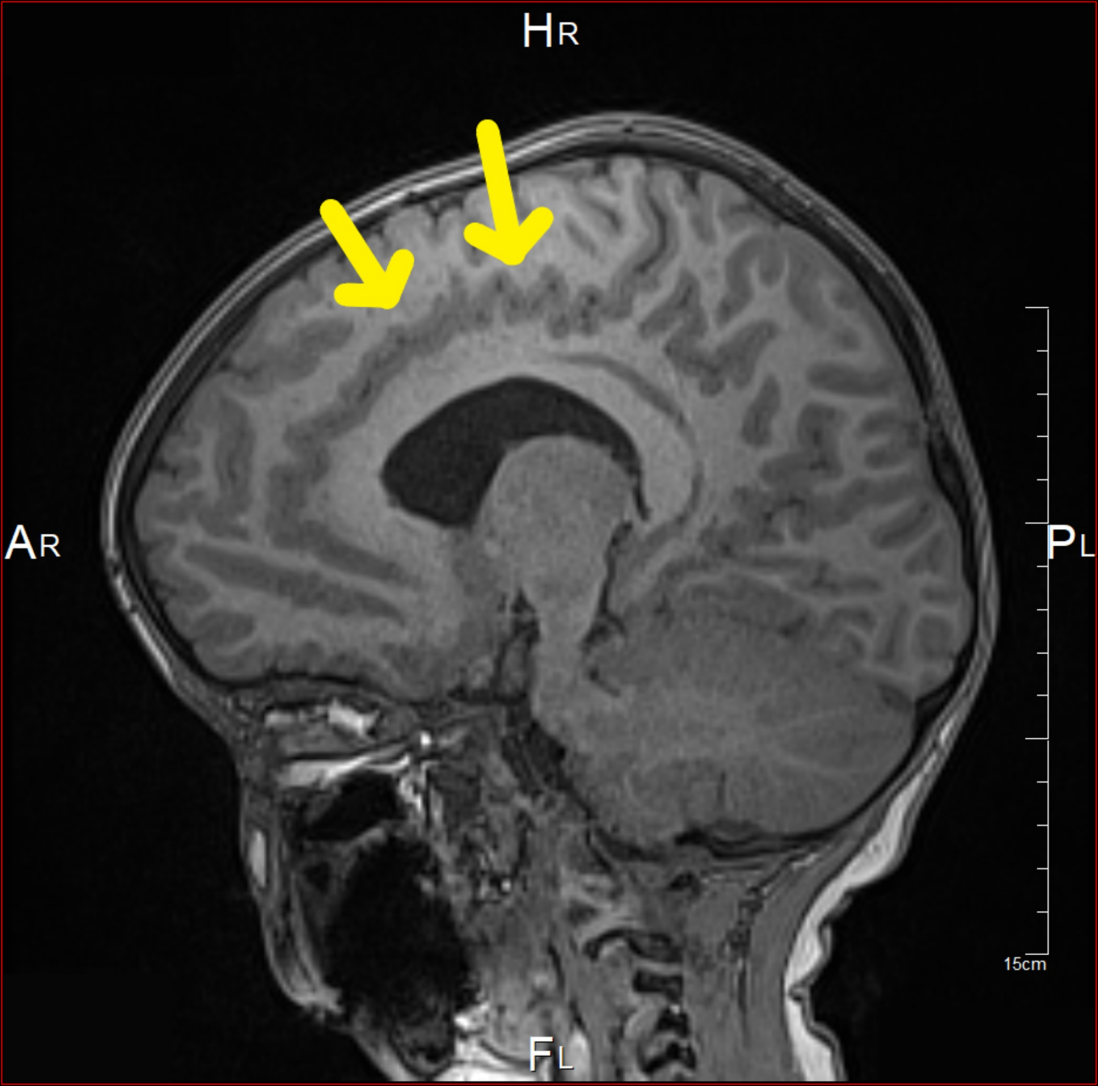
Brain MRI showing bifrontal and perisylvian polymicrogyria

**Figure 6:**
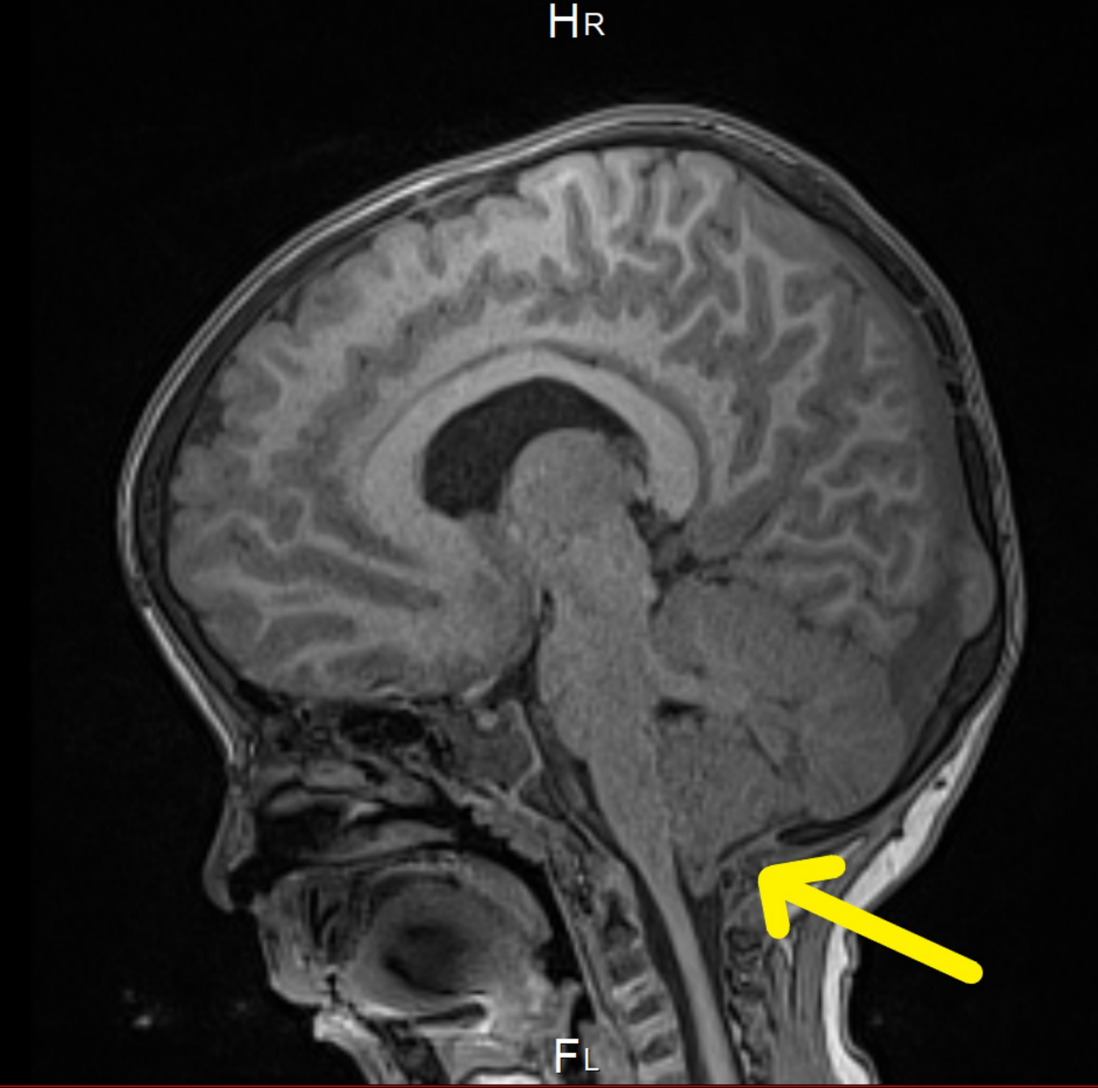
Brain MRI showing cerebellar tonsil ectopia with crowding of the posterior fossa

**Figure 7:**
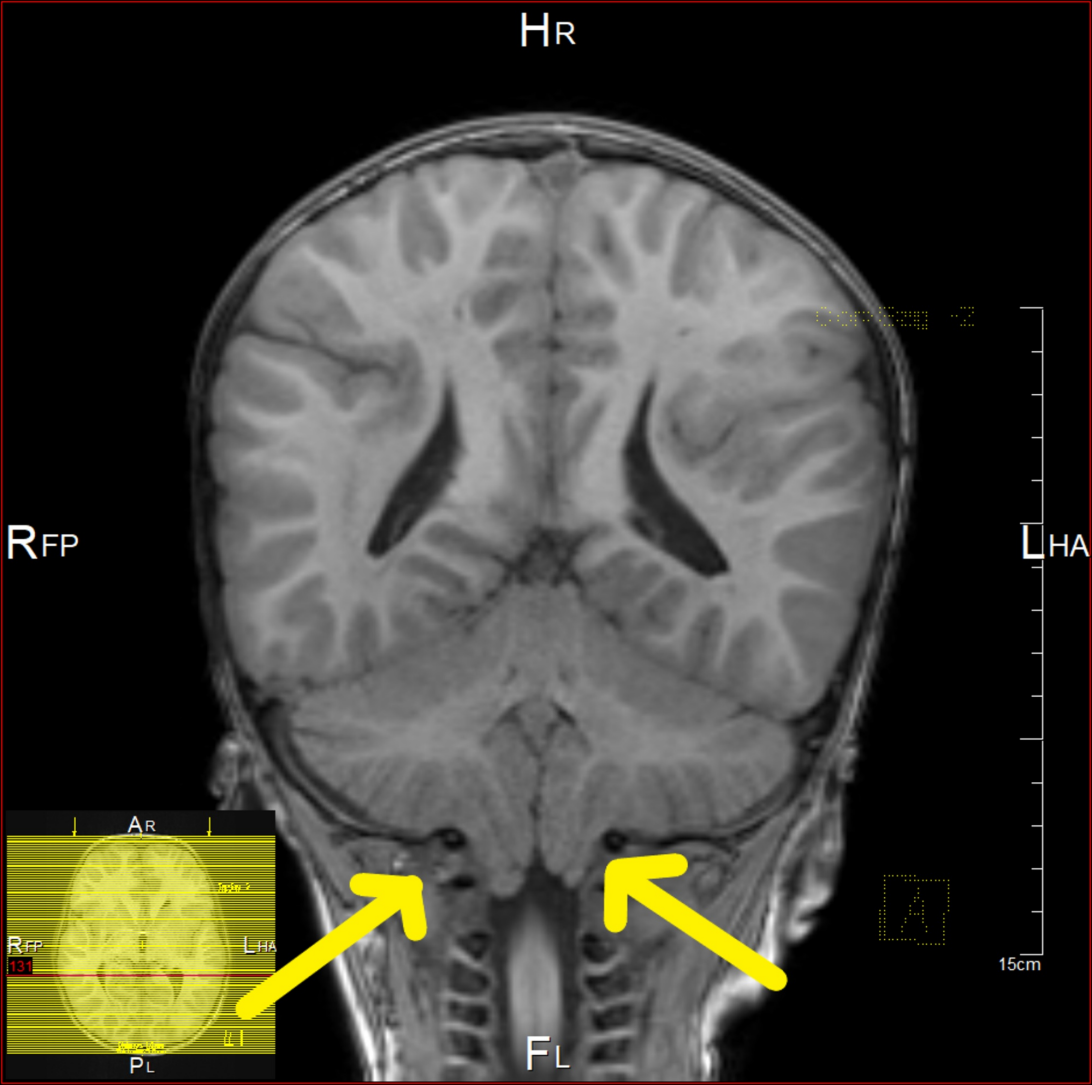
Brain MRI showing cerebellar tonsil ectopia with crowding of the posterior fossa

At the age of 41 months, MRI showed bifrontal and perisylvian polymicrogyria with areas of prominent Virchow Robin spaces and unchanged cerebellar tonsil ectopia with crowding of the posterior fossa. Bilateral peri-regional high T2 signal intensity was noted (Figures 4-7). Later, he was diagnosed by whole-exome sequencing (WES) as having changes in the PIK3CA gene, which results in PROS, namely, MCAP and mosaic confirmation. He had speech, language, and developmental delay and macrocephaly. The central nervous system (CNS) examination showed mild joint hyperlaxity, mild hypotonia in the upper and lower limbs, normal power, normal reflexes, and normal cranial nerves examination. Moreover, the patient was not found to have a headache, photophobia, vision problems, loss of consciousness, abnormal movements, seizure, or signs of increased intracranial pressure. The cardiac examination showed normal first and second heart sounds with no murmurs. On examination, there was no skin hyper/hypopigmentation nor were there any masses of growth. His head circumference had increased in size: 60 cm. He also had multiple dental caries and underwent full dental clearance with no complications. There was no evidence of regurgitation, choking, or feeding problems. The patient was started on the overgrowth protocol for PIK3CA, which includes alpha-fetoprotein and an abdominal US every three months until the age of eight years. The patient has followed up with the genetic, neurology, developmental, and general pediatrics clinics.

At the age of 43 months, another abdominal US was done to check for organomegaly, and it was unremarkable (Table 1). He was diagnosed with iron deficiency anemia and was prescribed ferrous sulfate 6 mg/kg/day. He was vitally stable, active, and playful. Regarding the milestones, he could not climb the stairs or eat by himself. He was able to walk with help. He preferred to watch things on his iPad and was on diapers. The mother noticed that when he got happy and excited, his hands fluttered. He had poor eye-to-eye contact with his mother or teacher at school. On examination, he was looking well; not in any pain or distress. He had positive subtle dysmorphic features in the form of low -set ears, macrocephaly with frontal bossing, depressed nasal bridge, and bilateral esotropia. His head circumference was 60 cm (above the 99th percentile). He had an unsteady gait but normal tone and power.

At the age of 46 months, the patient was reviewed in the developmental clinic. He started to say "ma" and "ba" only with unspecified one-syllable sounds. He could walk alone without support but could not run. He could go up and down the stairs without support. The patient could drink using glasses and cups but could not feed himself using spoons. He could not wave “bye-bye.” He liked repetitive actions, e.g., opening and closing doors multiple times and turning toy cars around multiple times. His mother was concerned that he lacked an awareness of danger. The mother said that he became more disciplined after attending one semester at a special center for speech therapy, physiotherapy, and occupational therapy. He is still on the overgrowth protocol for PIK3CA.

## Discussion

PROS is an umbrella that covers a wide spectrum of clinical phenotypes, ranging from isolated macrodactyly and truncal adipose overgrowth to extensive overgrowth of muscles, nerves, and adipose tissues in association with vascular malformation. Somatic PIK3CA mutations cause many overgrowth syndromes, including FAO, HHML, CLOVES syndrome, MCAP or M-CM, DMEG, and isolated macrodactyly [2].

MCAP is a rare genetic condition described by primary megalencephaly, prenatal overgrowth, asymmetrical brain, asymmetrical body, cutaneous vascular malformations, connective tissue, digital anomalies skin tissue dysplasia, subcutaneous dysplasia, affected joints, and brain cortex abnormalities such as polymicrogyria [4]. It is associated with various presentations and symptoms explaining the need for the number of investigations that have to be done to confirm the diagnosis, starting from simple blood tests to MRI and genetic testing. Our case is one of the first cases of MCAP in Saudi Arabia.

MCAP was named macrocephaly-cutis marmorata telangiectatica congenita (M-CMTC) in 1997, as the dermatological involvement was more prominent [5-6]. Later in 2007, the name M-CMTC changed to macrocephaly-capillary malformation syndrome (MCM), as the skin lesions were a type of capillary malformation that does not improve or worsen and is occasionally found with hypertrophic changes [7-8].

A few reports intended to outline some diagnostic criteria based on previous cases [9-10]. As new cases were studied, Mirzaa et al. shed light on perisylvian polymicrogyria and how common it is in numerous MCAP cases [4]. Thus, it is an essential part of the diagnostic process and an important finding in neuroimaging. In addition, they recommended the use of the term MCAP rather than MCM to reflect the large brain size, rather than relying on the head circumference.

Recent advances in genetic techniques used for diagnosis have shown the genes associated with the PI3K-AKT pathway in these patients [11-13]. PIK3A is a signaling enzyme that is critical for the growth, survival, and metabolism of most cells and is one of the most commonly mutated genes found in cancers. Rivière et al. reported that the mutations in the AKT3, PIK3R2, and PIK3CA genes are related to MCAP, hence suggesting the significance of PI3K/AKT signaling in vascular, limb, and brain growth [13]. Moreover, familial cases of MCAP were described, suggesting autosomal recessive inheritance or germline mosaicism. Mirzaa et al. also found that megalencephaly syndrome patients have de novo CCND2 mutations that lead to cyclin D caused either by CCND2 mutation or PI3K-AKT activation [4]. These advances can help identify new ways of treatment and make the process of diagnosis easier.

Neurological anomalies, such as cortical dysplasia, Arnold-Chiari malformation, and ventriculomegaly, are quite common in this syndrome [14-15]. These findings, along with some neurological manifestations, were reported as a result of the asymmetrical enlargement. Ventriculomegaly, cavum septum pellucidum or cavum vergae, cerebellar tonsillar herniation, cerebral and cerebellar asymmetry, thick corpus callosum, cortical dysplasia, and polymicrogyria are the main neuroimaging findings of this syndrome [7].

Unfortunately, sudden death seems to be a relatively common outcome for some reported cases. Ercan et al. described a case of a boy in Turkey who was born with tetralogy of Fallot (TOF) detected prenatally [16]. The child was diagnosed with MCAP and was found to have a thrombosed superior sagittal sinus on brain imaging. This child was the first case ever to have the syndrome along with TOF. Unfortunately, the child died at the age of six months. In addition, Harada et al. reported a case of a boy from Japan who was born at term via vacuum extraction with a poor Apgar score [17]. The child died suddenly while sleeping and autopsy CT did not reveal any abnormalities. A blood sample taken before death was tested for PI3K-AKT-mTOR pathway mutations, showing a heterozygous missense mutation in AKT3.

## Conclusions

This is a report of a 46-month-old boy, who was diagnosed with MCAP syndrome and is currently on overgrowth protocol. The management of patients with MCAP syndrome includes evaluation after the initial diagnosis, treatment of manifestations, and surveillance for potential complications, which may lead to death. However, at present, there is no curative treatment for patients with MCAP syndrome. Therefore, further studies and investigations of such syndrome are needed to aid in making a definitive classification and treatment of such complex and rare diseases.
